# Oral Health in Somalia: Unmasking a Neglected Dimension of Public Health

**DOI:** 10.1002/puh2.70406

**Published:** 2026-10-04

**Authors:** Ilyas Abdullahikhalif, Sharmake Gaiye Bashir, Aniso Mohamed Abdi, Yusuf Abdullahi Hubow, Abdirahman Mohamed Adan, Liibaan Abdulahi Sudi, Maryama Ahmed Ali, Nuradin Abdullahi Sheikh Rashid, Ahmed Abdinasir Abdulle

**Affiliations:** ^1^ Faculty of Health Sciences and Tropical Medicine Somali National University Mogadishu Somalia; ^2^ Center for Health Research and Innovation Somali National University Mogadishu Somalia; ^3^ Faculty of Health Sciences Salaam University Mogadishu Somalia

**Keywords:** health systems, noncommunicable diseases, oral health, Somalia, universal health coverage

## Abstract

Oral health is a fundamental but neglected component of public health, with important consequences for nutrition, communication, dignity, quality of life, and noncommunicable disease (NCD) prevention. In Somalia, oral health remains poorly integrated into health policy and service delivery within a context shaped by health‐system fragility, displacement, humanitarian pressures, and limited workforce capacity. This perspective synthesizes available Somalia‐specific evidence alongside modeled national estimates and relevant regional evidence to examine the burden, determinants, access barriers, and policy gaps surrounding oral health. It identifies major knowledge gaps, particularly the absence of comprehensive nationally representative oral health surveillance and limited evidence among rural, displaced, and other underserved populations. The article's contribution is to distinguish documented Somali evidence from regional inference while situating oral health within the country's broader universal health coverage, NCD, and humanitarian priorities. Strengthening surveillance, integrating essential oral health services into primary and humanitarian care, expanding preventive programs, and improving workforce and policy capacity are necessary to reduce avoidable oral disease and inequity in Somalia.

## 1 Introduction

Oral health is a core dimension of human well‐being, encompassing the ability to eat, speak, and socialize pain free; sustain self‐esteem and psychosocial functioning; and avoid systemic complications linked to chronic inflammation and infection [1]. Contemporary frameworks increasingly position oral conditions alongside other noncommunicable diseases (NCDs), recognizing shared behavioral and commercial risk factors and compelling evidence of bidirectional links with cardiovascular disease, diabetes, cancers, and mental health conditions [2]. Globally, oral diseases affect around 3.5–3.8 billion people and remain largely untreated, especially in low‑ and middle‑income countries [3].

Somalia exemplifies how fragile, conflict‐affected health systems magnify this burden. Protracted insecurity, displacement, underfunded infrastructure, and severe workforce shortages have produced substantial unmet health needs and financial barriers to care. Within this constrained system, oral health remains poorly integrated into health services, with limited policy support, inadequate surveillance, and restricted availability of preventive and restorative care [4].

Despite profound functional, psychosocial, and systemic consequences, oral health is systematically marginalized in Somalia's health agenda. This perspective argues that integrating oral health into NCD, universal health coverage, and humanitarian strategies is an urgent but overlooked public health priority requiring coordinated, multisectoral policy action [5].

## 2 The Burden of Oral Diseases in Somalia

Available evidence indicates a substantial burden of oral disease in Somalia, although nationally representative epidemiological data remain limited. The World Health Organization (WHO) Somalia Oral Health Country Profile reported modeled estimates for 2019 indicating untreated caries in deciduous teeth among 43.0% of children aged 1–9 years, untreated caries in permanent teeth among 33.7% of people aged 5 years and older, severe periodontal disease among 16.2% of people aged 15 years and older, and edentulism among 2.2% of adults aged 20 years and older [4]. These figures provide important indications of the likely national burden; however, they were derived from the Global Burden of Disease 2019 modeling framework rather than from a nationally representative clinical oral health survey conducted in Somalia [4]. They should, therefore, be interpreted as modeled national estimates rather than directly observed prevalence data.

Somalia‐specific empirical evidence remains geographically and demographically limited. A school‐based survey conducted in Hargeisa in December 2022 among 405 12‐year‐old children from 16 primary schools found a dental caries prevalence of 62.7%, with a mean decayed, missing, and filled teeth (DMFT) score of 1.7 and a Significant Caries Index (SiC) of 3.7 [6]. Only 14.7% of participants reported using dental care services during the preceding year [6]. Although this study provides valuable direct evidence of oral disease among Somali schoolchildren, its findings should not be generalized to the entire Somali population because the study was restricted to school‐attending 12‐year‐old children in Hargeisa.

Regional evidence provides additional context but cannot substitute for Somalia‐specific epidemiological surveillance. A systematic review and meta‐analysis covering East Africa estimated a pooled dental caries prevalence of 45.7% (95% CI: 38.0–53.4) [7]. However, the included studies showed very high between‐study heterogeneity (*I* ^2^ = 99.55%), indicating substantial variation across countries, study populations, age groups, dentition types, socioeconomic conditions, and settings [7]. Consequently, pooled East African estimates should be used to contextualize Somalia's oral health situation rather than interpreted as estimates of national prevalence. Oral cancer represents an additional but insufficiently characterized component of Somalia's oral health burden. The WHO Somalia Oral Health Country Profile estimated 175 new cases of lip and oral cavity cancer in 2020, corresponding to an age‐standardized incidence rate of 2.0 per 100,000 population [4]. However, these estimates should be interpreted cautiously because Somalia lacks comprehensive population‐based cancer surveillance. Early detection also remains a concern because oral health screening was reported as unavailable in public‐sector primary care facilities in the WHO Somalia profile [4]. Strengthening cancer surveillance and oral health monitoring systems is necessary to improve assessment of oral cancer burden in Somalia.

The scarcity of nationally representative oral health data is itself an important public health gap. Available evidence does not provide comprehensive population‐based estimates of dental caries, periodontal disease, tooth loss, treatment needs, and oral health inequalities across Somalia's different regions, age groups, rural and urban populations, and displaced communities. Data generation in fragile and conflict‐affected settings is also challenging because insecurity, population mobility and displacement, disrupted health services, resource constraints, and difficulties reaching geographically dispersed populations can impede representative surveys and routine health surveillance [8, 9]. Strengthening standardized national oral health surveillance is, therefore, necessary to establish the magnitude and distribution of oral diseases in Somalia and to reduce dependence on modeled and regional estimates.

Somalia‐specific evidence also demonstrates important gaps in oral health policy and service delivery. The WHO Somalia Oral Health Country Profile reported that, as of 2021, Somalia had no national oral health policy, strategy, or action plan recorded as operational or under development and no dedicated staff for oral health working on NCDs within the Ministry of Health [4]. The same profile reported that oral health screening for early disease detection, urgent treatment for emergency oral care and pain relief, and basic restorative dental procedures were unavailable in public‐sector primary care facilities [4]. However, the burden of oral disease in Somalia cannot be explained only by limited epidemiological data or service availability. It is also shaped by interacting socioeconomic, behavioral, commercial, and health‐system conditions that influence exposure, prevention, and access to care.

## 3 Determinants of Oral Health Inequities in Somalia

Oral health disadvantage in Somalia emerges from interconnected socioeconomic, behavioral, commercial, and health‐system conditions shaped by the country's wider social and political environment.

Socioeconomic inequalities position poor, displaced, and low‑education households at greatest risk. Across African and Middle East settings, lower income, unemployment, and low schooling are consistently associated with worse oral status, less preventive care, and lower health literacy, with oral diseases acting as a “sensitive marker” of deprivation [10]. In slum and disadvantaged communities, caries and periodontal disease cluster in low‑SES groups who rely on self‑care and inappropriate cleaning tools and seek dental care only when pain is severe [10].

These gradients are amplified by health system limitations. African countries report extremely low dentist densities, severe maldistribution favoring urban centers, minimal mid‑level cadres, and chronic underfunding of oral health, leaving rural and poor populations with the highest unmet need [10]. Fragile and conflict‑affected settings face further disruption of infrastructure and workforce, with oral care often absent from primary care and emergency responses [9].

Behavioral and lifestyle patterns emerge within this structural vacuum. In multiple African countries, infrequent toothbrushing, low toothpaste use, high sugar intake, and reliance on traditional remedies are common, especially among the poor and rural [11]. Widespread tobacco use and regionally entrenched khat chewing are linked to mucosal lesions, periodontal problems, prosthetic failures, and broader nutrition risks that compound oral vulnerability [12].

Tobacco use is an established modifiable risk factor for oral malignancy and should, therefore, form part of integrated oral health and NCD prevention strategies [2]. Khat chewing is also relevant to the regional risk environment because prolonged exposure has been associated with oral mucosal abnormalities [13]. More recently, a case‐control study among 97 male participants attending two cancer clinics in Hargeisa reported associations between greater frequency and amount of khat chewing and upper digestive tract cancers, which included oral cavity and esophageal cancers [14].

Finally, commercial and environmental drivers, including aggressive marketing and easy availability of sugar‑rich foods, tobacco, and other unhealthy commodities in LMICs under weak regulatory regimes, disproportionately harm poorer populations and fuel rising oral disease burdens [2]. Together, these forces reinforce unequal exposure to oral disease and unequal access to prevention and treatment, particularly among disadvantaged populations.

## 4 Oral Health in Somalia's Humanitarian and Displacement Context

The structural, socioeconomic, and health‐system determinants described above are further intensified by Somalia's prolonged humanitarian crisis, where conflict, displacement, climate shocks, and constrained health services shape both exposure to oral disease and the ability to obtain care. Protracted conflict, recurrent drought, food insecurity, population displacement, and constrained humanitarian financing continue to place substantial pressure on already limited health services. In 2026, an estimated 4.8 million people were identified as requiring humanitarian assistance [15]. Displacement is particularly important: as of 30 June 2026, Somalia hosted approximately 3.3 million internally displaced persons (IDPs), in addition to 32,966 refugees and asylum‐seekers [16]. These populations frequently live in settings where competing health priorities, mobility, poverty, and limited availability of health services can make continuity of preventive and curative care difficult.

Despite the scale of displacement, direct epidemiological evidence on oral health among Somali IDPs, refugees, and camp‐based populations remains extremely limited. Consequently, the prevalence of dental caries, periodontal disease, oral infections, and unmet dental treatment needs among these populations cannot currently be quantified with confidence. Evidence from fragile and conflict‐affected settings nevertheless indicates that disrupted health infrastructure, shortages of trained personnel, insecurity, financial barriers, and displacement can further restrict access to oral healthcare [9]. In Somalia, these structural conditions may contribute to delayed presentation and reliance on emergency or pain‐driven care; however, dedicated studies among displaced populations are required before the magnitude of these effects can be established.

Humanitarian health delivery platforms provide an important opportunity to reduce this gap. Mobile clinics and outreach services are already used to reach populations affected by conflict, displacement, and climate‐related emergencies, and mobile‐clinic quality tools have been piloted through the Somalia Health Cluster [17]. However, available evidence does not indicate that essential oral healthcare is routinely incorporated into humanitarian health packages in Somalia. A feasible initial approach would be to integrate basic oral health promotion, recognition of urgent dental infections and oral lesions, appropriate pain management within provider competencies, and referral pathways into primary healthcare, mobile health teams, nutrition programs, and other humanitarian services serving displaced and hard‐to‐reach communities.

International agencies, national and international nongovernmental organizations, the Ministry of Health, and Health Cluster partners could, therefore, play complementary roles in incorporating oral health into humanitarian health responses. Rather than establishing parallel dental programs, oral health could be progressively embedded within existing community health, mobile‐clinic, primary‐care, nutrition, and referral platforms. Such integration would extend essential preventive and emergency oral healthcare to populations that are otherwise difficult to reach while recognizing that comprehensive dental treatment requires appropriately trained personnel, equipment, infection‐control systems, and reliable referral mechanisms. Within this context, oral health outcomes in Somalia are shaped by interacting structural, humanitarian, health‐system, behavioral, and access‐related pathways rather than by isolated clinical factors alone. The conceptual framework presented in Figure 1 illustrates these relationships by showing how broader vulnerabilities influence service capacity, care‐seeking pathways, oral disease outcomes, and wider social and economic consequences.

**FIGURE 1:**
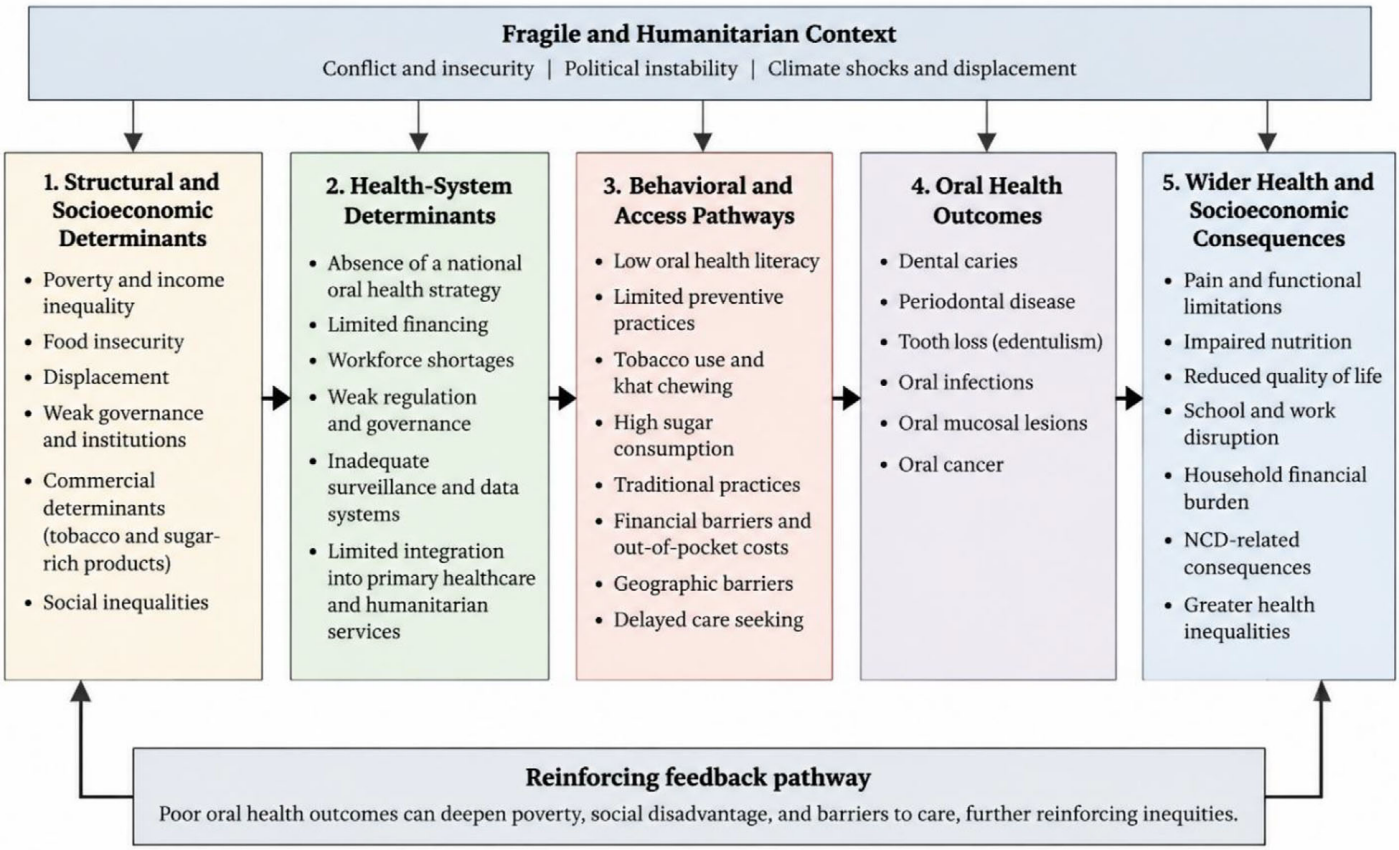
Integrated social–ecological framework of oral health inequities in Somalia: Pathways from structural vulnerability to oral health outcomes and socioeconomic consequences. NCD, noncommunicable disease.

## 5 Barriers to Accessing Oral Healthcare

Access to dental care in Somalia is constrained by overlapping financial, geographic, service‐delivery, and sociocultural barriers. Across low‑income countries, oral health services are minimally funded, predominantly private, and heavily reliant on out‑of‑pocket payments, with expenditures largely from domestic private sources and tooth extraction as the dominant covered intervention [2]. In Somalia specifically, the absence of public oral services and subsidies means only those who can afford expensive private care receive treatment, whereas cost and low perceived value of oral health drive delayed attendance and underutilization [18].

Severe workforce shortages and maldistribution further constrain access. Africa hosts only about 1% of the world's dentists, with very low dentist‐to‐population ratios and marked urban–rural inequities [19]. Somali practitioners report a shortage of dental professionals, unlicensed “quacks,” and a lack of integration of oral health into primary care, leaving rural and marginalized groups particularly underserved [18]. Weak integration mirrors broader global patterns where oral health is treated as an “optional” add‑on, hindered by poor infrastructure, fragmented systems, and limited training of non‐dental providers [20].

Sociocultural barriers, including low literacy, dental fear, stigma, and reliance on self‑medication or traditional remedies reinforce structural exclusion. Studies from Africa and Asia describe pain‑driven, delayed care; fears of injections and procedures; shame; and widespread self‐treatment that worsens disease severity before presentation [21]. In Somalia's fragile, impoverished context, these interacting barriers systematically delay care‑seeking, entrench advanced disease, and reproduce inequities in who can maintain basic oral function and dignity [18]. When these barriers remain unresolved, oral diseases are more likely to progress without timely prevention or treatment, increasing their consequences for individual well‐being, household finances, education, and productivity.

## 6 Public Health and Socioeconomic Impact

Untreated oral disease in Somalia has consequences extending beyond the mouth, affecting daily functioning, education, productivity, household finances, and general well‐being. Globally, untreated oral diseases cause unremitting pain, sepsis, functional limitations, and reduced quality of life, with major disruption of eating, sleeping, speaking, and social interaction [2]. Children experience school difficulties, including problems with schoolwork and, in many settings, increased absenteeism when oral pain and dysfunction are present [22]. Such impacts are not trivial inconveniences; they erode human capital formation, especially where education and employment opportunities are already fragile.

Indirectly, oral health neglect undermines productivity through lost work days and impaired performance, with studies documenting decreased work productivity and disruption of economic activities at household level [2]. At scale, oral diseases generate substantial economic losses: globally, oral conditions account for hundreds of billions of dollars annually in treatment costs and productivity losses, with particularly stark underinvestment and high unmet need in low‑income settings [2]. For Somali households reliant on out‑of‑pocket payments and informal work, even modest dental expenditures or time off work can be economically destabilizing.

Clinically, periodontal disease and other oral conditions are strongly associated with diabetes, cardiovascular disease, obesity, and other NCDs through shared inflammatory, nutritional, and behavioral pathways [23]. Poor systemic control worsens oral disease, whereas oral inflammation can aggravate metabolic and vascular risk, creating a bidirectional, syndetic relationship that amplifies morbidity and costs [23].

These consequences make oral healthcare relevant to Somalia's broader goals of NCD prevention, financial protection, and human development.

## 7 Policy Gaps and Missed Opportunities

Gaps in Somalia's dental services are rooted largely in weaknesses in policy, financing, governance, surveillance, and service organization rather than individual behavior alone. Across low‑income settings, oral health receives little political and resource commitment, with many countries showing no or only partial integration of oral health into national policies and UHC agendas, minimal public spending, and service packages dominated by tooth extraction rather than prevention [2]. In the WHO African region, frequently cited barriers include absent or weak national oral health policies, poor governance, lack of health information systems, and inadequate integration of oral health within overarching health systems [24]. Fragile and conflict‑affected states face additional strains: competing priorities, resource depletion by crises, and workforce loss further sideline oral health in planning and financing decisions [9].

Limited surveillance is both a symptom and driver of neglect. Global analyses highlight the absence of robust oral health indicators and monitoring frameworks, undermining the inclusion of oral health in UHC tracking and national decision‐making [25]. Exclusion of oral health from primary care and UHC benefit packages leads to high out‑of‑pocket payments, low utilization, and large rich–poor gaps in unmet need, directly contradicting UHC's equity goals [25].

These shortcomings highlight the need for stronger governance, reliable surveillance, and formal incorporation of essential dental services into primary healthcare and national NCD strategies [2]. Addressing these interconnected challenges requires moving beyond identifying oral health neglect toward practical strategies that strengthen prevention, service delivery, workforce capacity, and financial protection.

## 8 Way Forward: Strategic Recommendations

A phased reform agenda could progressively strengthen prevention, service delivery, workforce capacity, and financial protection for dental care in Somalia. First, integration into primary healthcare should focus on equipping nurses, midwives, and community health workers to deliver basic oral health promotion, screening, fluoride varnish, and timely referral, using short standardized curricula and job aids adapted from African integration experiences and WHO frameworks [26]. Mobile and outreach services, including tele dentistry, can extend coverage to displacement camps and rural areas in line with fragile state innovations [20]. Second, workforce development and task shifting should prioritize mid‐level providers and community‐based cadres, supported by clear scopes of practice, supervision, and incentives for rural deployment [20]. Regional training partnerships and competency‐based curricula that embed prevention and community rotations can gradually rebalance the urban, treatment‐focused model [20]. Third, population level prevention is essential. School‐based oral health programs combining skills‐based education, daily supervised toothbrushing with fluoridated toothpaste, and restriction of sugary snacks have reduced caries and plaque in low resource settings and are feasible where schools already serve as health platforms [27]. Community campaigns using existing maternal child and social programs can strengthen oral health literacy and address myths [28]. Fourth, financial protection and policy levers are critical for equity. Inclusion of a basic package of preventive and emergency oral services within essential benefits, with targeted subsidies for poor and displaced households, aligns with emerging universal oral health coverage models [8]. Finally, regulatory policies, such as taxation of sugar‐sweetened beverages and measures to reduce children's sugar exposure, can modestly lower sugar intake and caries burden while generating earmarked revenue for oral health promotion [29]. Taken together, these measures provide a practical pathway for expanding access to preventive and essential dental services while reducing avoidable disparities.

## 9 Conclusion

Oral diseases represent an important but insufficiently addressed component of Somalia's public health burden. Their consequences extend beyond pain and infection to nutrition, education, productivity, household expenditure, and the management of NCDs. These challenges are intensified by limited‐service availability, workforce shortages, humanitarian pressures, and persistent socioeconomic disparities. Integrating essential dental services into primary and humanitarian care, strengthening surveillance, expanding preventive programs, and developing an appropriately trained workforce could substantially improve access and reduce avoidable disease. Progress will require coordinated action across health authorities, professional institutions, humanitarian partners, communities, and other relevant sectors. Addressing these gaps would contribute not only to better dental outcomes but also to greater health equity and stronger health‐system resilience in Somalia.

## Author Contributions

Ilyas Abdullahi khalif conceptualized this topic. Ahmed Abdinasir Abdulle and Nuradin Abdullahi Sheikh Rashid supervised this project. Abdirahman Mohamed Adan and Yusuf Abdullahi Hubow conducted literature review. Liibaan Abdulahi Sudi, Aniso Mohamed Abdi, and Maryama Ahmed Ali wrote the first draft. Sharmake Gaiye Bashir revised and edited this manuscript. All authors approved the final version of the manuscript.

## Funding

The authors have nothing to report.

## Conflicts of Interest

The authors declare no conflicts of interest.

## Data Availability

Data sharing is not applicable to this article as no datasets were generated or analyzed during the current study.

## References

[1] A. Barranca‐Enríquez and T. Romo‐González , “Your Health Is in Your Mouth: A Comprehensive View to Promote General Wellness,” Frontiers in Oral Health 3 (2022): 971223, 10.3389/FROH.2022.971223. PMC9515542 36186536

[2] M. A. Peres , L. M. D. Macpherson , R. J. Weyant , et al., “Oral Diseases: A Global Public Health Challenge,” Lancet 394 (2019): 249–260, 10.1016/S0140-6736(19)31146-8. 31327369

[3] E. Bernabe , W. Marcenes , C. R. Hernandez , et al., “Global, Regional, and National Levels and Trends in Burden of Oral Conditions From 1990 to 2017: A Systematic Analysis for the Global Burden of Disease 2017 Study,” Journal of Dental Research 99 (2020): 362–373, 10.1177/0022034520908533. PMC7088322 32122215

[4] World Health Organization , “Oral Health Somalia 2022 Country Profile,” World Health Organization, accessed August 19, (2026), https://www.who.int/publications/m/item/oral‐health‐som‐2022‐country‐profile.

[5] S. R. Samuel , S. Acharya , and J. C. Rao , “School Interventions–Based Prevention of Early‐Childhood Caries Among 3–5‐Year‐Old Children From Very Low Socioeconomic Status: Two‐Year Randomized Trial,” Journal of Public Health Dentistry 80 (2020): 51–60, 10.1111/JPHD.12348. 31710096

[6] A. E. Dahroug , E. K. Heen , M. A. Hussein , and A. A. Madar , “Dental Caries Status and Related Factors Among 12‐Year‐Old Somali School Children in Hargeisa,” Community Dentistry and Oral Epidemiology 52 (2024): 861–870, 10.1111/CDOE.12990. 38923041

[7] A. Teshome , A. Muche , and B. Girma , “Prevalence of Dental Caries and Associated Factors in East Africa, 2000–2020: Systematic Review and Meta‐Analysis,” Frontiers in Public Health 9 (2021): 645091, 10.3389/FPUBH.2021.645091. PMC8116500 33996722

[8] Y. Luan , D. Sardana , A. Jivraj , et al., “Universal Coverage for Oral Health Care in 27 Low‐Income Countries: A Scoping Review,” Global Health Research and Policy 9 (2024): 34, 10.1186/S41256-024-00376-9. PMC11384684 39252095

[9] B. Bogale , S. Scambler , A. N. Mohd Khairuddin , and J. E. Gallagher , “Oral Health System Strengthening in Fragile and Conflict‐Affected States: A Systematic Review,” Journal of Global Health 14 (2024): 04132, 10.7189/JOGH-14-04132. PMC11190633 38902973

[10] E. O. Ogunbodede , I. A. Kida , H. S. Madjapa , et al., “Oral Health Inequalities Between Rural and Urban Populations of the African and Middle East Region,” Advances in Dental Research 27 (2015): 18–25, 10.1177/0022034515575538. 26101336

[11] D. O. Shomuyiwa and G. Bridge , “Oral Health of Adolescents in West Africa: Prioritizing Its Social Determinants,” Global Health Research and Policy 8 (2023): 28, 10.1186/S41256-023-00313-2. PMC10355062 37468980

[12] A. Bennani and S. I. Mohamed , “Effect of Khat Consumption on Oral Health: Study Carried out in Djibouti City,” Oral Health and Dental Science 5 (2021): 1–8, 10.33425/2639-9490.1077.

[13] A. M. Ali , N. Huang , and J. J. Liu , “Khat Chewing and Its Association With Oral/Esophageal Squamous Cell Carcinoma and Oral White Lesions: A Systematic Review and Meta‐Analysis,” Public Health 252 (2026): 106177, 10.1016/j.puhe.2026.106177. 41616555

[14] A. M. Ali , M. N. Mutuku , A. Hashi , and O. M. Muhumed , “Erratum: Relationship Between Khat Chewing and Upper Digestive Tract Cancers Among Male Patients in Hargeisa: A Preliminary Case‐Control Study,” Ecancermedicalscience 20 (2026): 2094, 10.3332/ECANCER.2026.2094. PMC13161583 42131888

[15] OCHA , “Somalia Humanitarian Needs and Response Plan 2026,” OCHA, accessed August 19, 2026, World Health Organization (WHO), https://www.unocha.org/publications/report/somalia/somalia‐humanitarian‐needs‐and‐response‐plan‐2026.

[16] “Document—Somalia Refugees and Internal Displacement_June 2026,” accessed August 19, 2026, https://data.unhcr.org/en/documents/details/123401.

[17] “Advancing Quality of Care in Mobile Clinics: A Global Step Forward,” accessed August 19, 2026, World Health Organization (WHO), https://healthcluster.who.int/newsroom/news/item/05‐11‐2025‐advancing‐quality‐of‐care‐in‐mobile‐clinics‐a‐global‐step‐forward.

[18] S. Gedi , A. M. Hassan , M. M. Dahir , et al., “Challenges in Oral Health Practice in Somalia: A Call for Regulatory Strengthening and Inclusion Into Primary Healthcare Services,” BMC Oral Health 24 (2024): 1440, 10.1186/S12903-024-05221-6. PMC11603915 39604947

[19] J. E. Gallagher and L. Hutchinson , “Analysis of Human Resources for Oral Health Globally: Inequitable Distribution,” International Dental Journal 68 (2018): 183–189, 10.1111/IDJ.12349. PMC9378901 29297930

[20] M. O. Foláyan , A. G. Ishola , A. Bhayat , M. El Tantawi , and N. Ndembi , “Strengthening Health Systems to Tackle Oral Diseases in Africa: Africa Centers for Disease Control and Prevention's Role,” Front Public Health 13 (2025): 1539805, 10.3389/FPUBH.2025.1539805. PMC11798923 39916711

[21] S. O. Gbadebo , P. U. Okereke , G. A. Oke , and O. O. Dosumu , ““Since I Never Felt Pain, It Didn't Occur to Me to Visit a Dental Clinic”: A Qualitative Exploration of Social Perceptions and Influencers of Oral Care‐Seeking Behaviour Among Adult Nigerians,” BMC Oral Health 25 (2025): 1196, 10.1186/S12903-025-06634-7. PMC12275304 40682060

[22] R. R. Ruff , S. Senthi , S. R. Susser , and A. Tsutsui , “Oral Health, Academic Performance, and School Absenteeism in Children and Adolescents,” Journal of the American Dental Association 150 (2019): 111–121.e4, 10.1016/J.ADAJ.2018.09.023. 30473200

[23] N. T. Hashim , R. Babiker , V. Padmanabhan , et al., “The Global Burden of Periodontal Disease: A Narrative Review on Unveiling Socioeconomic and Health Challenges,” International Journal of Environmental Research and Public Health 22 (2025): 624, 10.3390/IJERPH22040624. PMC12027323 40283848

[24] M. Mohammadpour , P. Bastani , D. Brennan , A. Ghanbarzadegan , and J. Bahmaei , “Oral Health Policymaking Challenges in Iran: A Qualitative Approach,” BMC Oral Health 20 (2020): 158, 10.1186/S12903-020-01148-W. PMC7268740 32487152

[25] R. R. Basapathy and M. R. Mathur , “Strengthening Governance for Universalising Primary Oral Health Care: Perspectives From Karnataka, India,” Wellcome Open Research 10 (2025): 305, 10.12688/wellcomeopenres.24152.1. PMC12595295 41208825

[26] M. Prasad , C. Manjunath , A. Murthy , A. Sampath , S. Jaiswal , and A. Mohapatra , “Integration of Oral Health Into Primary Health Care: A Systematic Review,” Journal of Family Medicine and Primary Care 8 (2019): 1838, 10.4103/JFMPC.JFMPC_286_19. PMC6618181 31334142

[27] P. Akera , S. E. Kennedy , R. Lingam , M. J. Obwolo , A. E. Schutte , and R. Richmond , “Effectiveness of Primary School‐Based Interventions in Improving Oral Health of Children in Low‐ and Middle‐Income Countries: A Systematic Review and Meta‐Analysis,” BMC Oral Health 22 (2022): 264, 10.1186/S12903-022-02291-2. PMC9245251 35768801

[28] P. E. Petersen , R. J. Baez , and H. Ogawa , “Global Application of Oral Disease Prevention and Health Promotion as Measured 10 Years After the 2007 World Health Assembly Statement on Oral Health,” Community Dentistry and Oral Epidemiology 48 (2020): 338–348, 10.1111/CDOE.12538. PMC7496398 32383537

[29] M. Hajishafiee , K. Kapellas , S. Listl , M. Pattamatta , A. Gkekas , and P. Moynihan , “Effect of Sugar‐Sweetened Beverage Taxation on Sugars Intake and Dental Caries: An Umbrella Review of a Global Perspective,” BMC Public Health [Electronic Resource] 23 (2023): 986, 10.1186/S12889-023-15884-5. PMC10224604 37237341

